# Mixed-methods evaluation of mental healthcare integration into tuberculosis and maternal-child healthcare services of four South African districts

**DOI:** 10.1186/s12913-019-3912-9

**Published:** 2019-01-31

**Authors:** Kathryn L. Lovero, Samantha L. Lammie, André van Zyl, Sharon N. Paul, Phuti Ngwepe, Jennifer J. Mootz, Catherine Carlson, Annika C. Sweetland, Rachel C. Shelton, Milton L. Wainberg, Andrew Medina-Marino

**Affiliations:** 1 New York State Psychiatric Institute, Department of Psychiatry, Columbia University Vagelos College of Physicians and Surgeons, 1051 Riverside Drive #24, New York, NY 10032 USA; 2 Emory University School of Medicine, 1648 Pierce Dr NE, Atlanta, GA 30307 USA; 3 Foundation for Professional Development, 173 Mary Rd, Die Wilgers, Pretoria, 0184 South Africa; 4 School of Social Work, University of Alabama, 3026 Little Hall, Box 870314, Tuscaloosa, AL 35487-0314 USA; 5 Department of Sociomedical Sciences, Mailman School of Public Health, Columbia University, 722 W. 168th St. #941, New York, NY 10032 USA

**Keywords:** Mental health, Mental disorders, Primary care, Healthcare integration, Health systems, South Africa, Sub-Saharan Africa, Mixed-methods, Implementation

## Abstract

**Background:**

The South African National Mental Health Policy Framework and Strategic Plan 2013–2020 was adopted to address the country’s substantial burden and inadequate treatment of mental illness. It outlines measures toward the goal of full integration of mental health services into primary care by 2020. To evaluate progress and challenges in implementation, we conducted a mixed-methods assessment of mental health service provision in tuberculosis and maternal-child healthcare services of four districts in South Africa.

**Methods:**

Forty clinics (ten per district) were purposively selected to represent both urban and rural locations. District-level program managers (DPMs) for mental health, tuberculosis, and maternal-child healthcare were qualitatively interviewed about district policy and procedures for management of mental illness and challenges in integrating mental health services into primary care. Clinic nurses and mental health practitioners (MHPs) completed a quantitative questionnaire to assess their engagement with stepped care for patients with mental illness. Qualitative and quantitative data were collected concurrently and compared to triangulate progress in implementation of integrated services.

**Results:**

A total of 59 nurses and 17 MHPs completed questionnaires, and nine DPMs were interviewed (total *n* = 85). DPMs indicated that nurses should screen for mental illness at every patient visit, although only 43 (73%) nurses reported conducting universal screening and 26 (44%) reported using a specific screening tool. For patients who screen positive for mental illness, DPMs described a stepped-care approach in which MHPs diagnose patients and then treat or refer them to specialized care. However, only 7 (41%) MHPs indicated that they diagnose mental illness and 14 (82%) offer any treatment for mental illness. Addressing challenges to current integration efforts, DPMs highlighted 1) insufficient funding and material resources, 2) poor coordination at the district administrative level, and 3) low mental health awareness in district administration and the general population.

**Conclusions:**

Though some progress has been made toward integration of mental health services into primary care settings, there is a substantial lack of training and clarity of roles for nurses and MHPs. To enhance implementation, increased efforts must be directed toward improving district-level administrative coordination, mental health awareness, and financial and material resources.

## Background

Mental and substance use disorders are the leading cause of years lived with disability worldwide and account for 7.4% of the total global health burden [1, 2]. Despite the substantial impact of mental illness, treatment rates worldwide are alarmingly low, and the mental health treatment gap is even more pronounced in low- and middle-income countries (LMICs) [3–5]. Integration of mental health services into primary care has been widely recognized as an evidence-based method to improve coverage for the treatment of mental illness in low-resource settings [6, 7]. However, emerging research indicates that efforts to implement integrated mental health services in primary care settings of LMICs have been hampered by a number of challenges, including severe financial constraints; inadequate training, support, and supervision for primary care providers of mental health services; uncoordinated or inconsistent referral pathways; and inadequate policy [8–15].

Like other LMICs, South Africa struggles with large numbers of individuals with untreated mental illness. Data from the first and only nationally representative psychiatric epidemiology study, conducted between 2003 and 2004, showed a 30.3% lifetime prevalence of common mental disorders among South Africans [16]. Moreover, 75% of respondents who had experienced mental illness in the past year had not received any treatment, likely owing to a significant shortage of trained personnel for mental health services in the public health sector [17]. More recent studies conducted at the provincial, district, and city level have documented continued inconsistent and irregular identification and treatment of mental disorders [18–20], and mental disorders are currently estimated to be the leading cause of years lived with disability in South Africa [21].

In 2013, the South African National Department of Health adopted its first national mental health policy, The National Mental Health Policy Framework and Strategic Plan 2013–2020 (the Strategic Plan) [22], which is aligned with the WHO Mental Health Action Plan [23]. By 2020, the Strategic Plan aims to have full integration of mental health assessment and management into all aspects of primary care, with an emphasis on tuberculosis (TB), HIV, and antenatal care services. Per the Strategic Plan, the implementation of integrated mental health services—including the establishment of routine screening and management of mental disorders in primary care, the development of routine referral pathways from primary care to specialist services, as well as the training of primary care providers—was to be coordinated at the district administrative level. However, since the adoption of the Strategic Plan, no formal evaluation has been conducted to assess the progress in implementation of integrated services within different South African districts.

We therefore conducted a mixed-methods evaluation of the integration of mental healthcare within four districts of South Africa to: 1) gain in-depth insight on stepped-care procedures designed by different districts for management of mental illness in primary care services; 2) determine the level to which integrated mental healthcare procedures have been implemented in primary care; and 3) identify challenges encountered in the district-level coordination of integration efforts. We focused specifically on the integration of mental health services into tuberculosis (TB) and maternal-child health (MCH) primary care services, as the Strategic Plan highlighted these services as a focus for integration efforts owing to the high prevalence of mental disorders among patients attending these services in South Africa [24–29].

## Methods

### Context

In the post-apartheid era, the South African National Department of Health has taken several steps to introduce policy that promotes deinstitutionalization and decentralization of mental healthcare [18, 19]. However, a nationwide situational analysis conducted in 2007 demonstrated that South Africa still relied heavily on psychiatric hospitals for the provision of mental healthcare [30]. Furthermore, efforts toward deinstitutionalization had not been equally met with development of community-based services, leaving many individuals without care [30].

In 2012, a series of Mental Health Summits were convened by the Minister of Health to develop consensus on national policy priorities for mental healthcare and address the countries large mental health treatment gap. Following this, the Strategic Plan was created, based on recommendations from the summits and from research on mental healthcare in South Africa [31]. One of the main objectives of the Strategic Plan is to “scale up decentralized integrated primary mental health services,” with the goal of including universal, routine screening and a stepped approach to management of mental disorders in primary care clinics.

To achieve this goal, the Strategic Plan calls for staff in primary care settings to receive training and supervision for basic mental health screening, diagnosis, and treatment as well as referral of complex cases. Specifically, it notes that key staff should be trained and supervised to use the *Primary Care 101 Guidelines* (PC101)—a South African clinical management tool that includes information on the screening, diagnosis, treatment, and referral of patients with mental illness, in addition to other illnesses [32]. The Strategic Plan does not outline the specific stepped approach that should be developed for integrated mental healthcare. Rather, it delegates to district level administrators the establishment of routine screening, diagnosis, and treatment in primary care; the development of referral pathways to specialist services; and the training of primary care providers on delivery of stepped mental health services.

### Study location

The study was conducted in four districts located in Mpumalanga, Gauteng, Limpopo, and Eastern Cape provinces of South Africa. In two districts (one located in each Eastern Cape and Mpumalanga provinces) we investigated integration into the TB program platform (TB Study Arm), and in another two districts (one located in each Gauteng and Limpopo provinces) we investigated integration into the maternal-child health program platform (MCH Study Arm). In consultation with administrators at the district health department, purposive recruitment and selection of ten clinics per district was carried out to include an equal representation of urban and rural clinics as well as distributed geographic locations throughout the district.

### Study design

We used a mixed-methods exploratory design in which the quantitative and qualitative data were collected concurrently and weighted equally (QUANT+QUAL) [33] to investigate implementation of integrated care from the administrative and provider perspective. We collected qualitative data from district administrators to explore in-depth the procedures for stepped-care management of mental illness implemented within primary care clinics of their district, as well as the perceived challenges they encountered in implementing integrated mental health services. We collected quantitative data using a structured questionnaire to obtain information on the training providers had received for management of mental illness, their experiences managing mental illness, and the specific aspects of stepped care (screening, diagnosis, treatment, referral) they performed.

### Study population

Per the Strategic Plan, the development of routine screening and management of mental disorders in primary care, routine referral pathways from primary care to specialist services, as well as the training of primary care providers was to be coordinated at the district administrative level. Therefore, the study team conducted semi-structured interviews with district-level program managers (DPMs) in each of the four study districts (Table 1). In every district, the DPM in charge of the mental health services portfolio was interviewed. In the district located in the Eastern Cape province, this was the general health program manager; in all other districts, this was the DPM explicitly in charge of mental health. Additionally, we sought to interview the TB program manager of districts in the TB Study Arm and the MCH program manager of districts in the MCH Study Arm. In the district located in the Eastern Cape province, the district TB program manager was not available, so a sub-district TB program manager was interviewed. In the district located in the Mpumalanga province, the District Director of Primary Healthcare also indicated (s)he participates in TB service programming activities, and thus was also interviewed. Table 2 provides professional profiles of the DPMs interviewed (*n* = 9).

**Table 1:** Study districts and participants

	MCH Study Arm	TB Study Arm
Province of Study District	Gauteng and Limpopo	Eastern Cape and Mpumalanga
Clinics	20 (10/District)	20 (10/District)
*Qualitative sample*		
District program managers (DPM)	4	5
*Quantitative sample*		
Nurses	39	20
Mental health practitioners (MHP)	9	8

**Table 2:** District program manager interview participants

Title	Study Arm	Interview ID
District Director of Primary Healthcare	TB	DPM 1
District Mental Health Program Manager	TB	DPM 2
District TB Program Manager	TB	DPM 3
District General Health Program Manager	TB	DPM 4
Sub-district TB Program Manager	TB	DPM 5
District Mental Health Program Manager	MCH	DPM 6
District Maternal and Child Health Program Manager	MCH	DPM 7
District Mental Health Program Manager	MCH	DPM 8
District Maternal and Child Health Program Manager	MCH	DPM 9

We also administered quantitative questionnaires to providers most likely to be involved at the key stages in stepped-care management: screening, diagnosis, and treatment in primary care, as well as referral to specialist services. Nurses are the first, and often only, provider in primary care clinics. Therefore, the study team administered structured questionnaires to one or two nurses—TB nurses in the TB Study Arm; MCH nurses in the MCH Study Arm—per clinic. In total, 59 nurses were included: 39 (66.1%) MCH nurses and 20 (33.9%) TB nurses (Table 1). Mental health practitioners (MHPs) are physicians, nurses, occupational therapists, psychologists, and social workers trained to provide prescribed mental healthcare, treatment, and rehabilitation services in primary care clinics. Therefore, the study team also sought to administer structured questionnaires to one MHP per clinic. However, not all clinics had a MHP available for participation in the study, and thus 17 MHPs were included in total: nine (52.9%) at clinics in the TB Study Arm and eight (47.1%) at clinics in the MCH Study Arm (Table 1). No nurses or MHPs who were invited to the study declined to participate.

### Data collection

Trained study staff enrolled participants and collected both quantitative and qualitative data between November 2016 and July 2017. Prior to enrollment, the study was described to all participants, and written informed consent was obtained. All interviews were conducted in English; all interviewees self-reported to be native or proficient in English.

### Qualitative data collection

Semi-structured interviews conducted with DPMs lasted approximately 1 h. DPMs were asked their perspectives about: 1) screening, diagnostic, and referral processes for identification and management of mental illness in primary care; 2) mental health training for primary care providers; 3) impact of mental health integration at primary care clinics; 4) challenges with providing integrated mental health services; and 5) future needs for successful integration of mental healthcare. Interview guides were used by study staff for all DPM interviews, and 77.8% (*n* = 7) of DPMs agreed to have their interviews recorded. Extensive notes were taken using the hard copy interview guide for all DPM interviews.

### Quantitative data collection

The study team administered questionnaires, based on a survey used in previous studies to evaluate integration of mental healthcare into primary care services [34], face-to-face with nurses and MHPs at each clinic. Nurses in both TB and MCH services were asked about: 1) training on mental health assessment; 2) procedures and tools used in screening for mental illness; 3) referral processes for patients with mental illness; and 4) experiences managing mental healthcare. MHPs in both TB and MCH services were asked about: 1) procedures and tools used in screening and diagnosis of mental illness; 2) referral processes for patients with mental illness; and 3) treatments for mental illness they provide. Nurse and MHP responses were recorded by study team members using digital questionnaires designed for the study on the REDCap data collection platform—a secure, web-based application for research data capture and management [35].

### Data analysis

Audio recordings of DPM interviews were transcribed verbatim by two trained study team members, and all transcripts were reviewed against audio recordings for accuracy. We conducted a content analysis [36–38] to explore, characterize, and code qualitative data. An initial codebook was created by the study team using interview guides, literature, and a preliminary analysis of nurse and MHP questionnaire responses gathered during the first half of the study. Two study team members, who also performed interviews with DPMs, conducted line-by-line coding of individual DPM transcripts. Discrepancies in coding were discussed with a third study team member after coding of each transcript and resolved through discussion and consensus. This process was iterated until the coding of the fifth manuscript, at which point coding agreement reached 80%. Line-by-line coding was then conducted on the remaining transcripts (*n* = 4) by a single study team member. Analysis of content was performed using code queries and code matrices in NVivo11 software [39]. Microsoft Excel was used to further explore text content and processes relating to the study aims. Descriptive data from nurse and MHP questionnaires were exported from REDCap and frequencies and percentages of responses were calculated in Stata/IC 14 (Statacorp, College Station, USA). Qualitative and quantitative data were compared after the completion of individual analysis of both to triangulate progress in implementation of integrated services.

## Results

### District manager description of integrated mental health service provision

Through qualitative interviews, we identified two themes related to DPM perceptions of how stepped-care management of mental illness should be provided within primary care clinics of their districts: (1) mental health screening should be conducted by nurses for all patients at primary healthcare facilities, though some acknowledge this is not universally implemented, and (2) mental healthcare referrals should be made within clinic to MHPs and/or to other facilities based on case severity and availability of mental health personnel within clinic. Additionally, we identified three themes that DPMs indicated as significant challenges to implementation of integrated care: (1) absence of coordination across health programs in district level administration, (2) lack of material and human resources, and (3) low mental health awareness in both the district administration and general population.

#### Universal screening procedures in primary healthcare

DPMs indicated that mental health screenings should be provided for all patients that come to the clinics. Nurses were the most common type of healthcare worker described as being responsible for mental health screening and were specifically expected to use the PC101 tool. Some DPMs also indicated that all professionals at the clinics should be screening patients.

“*Nurses has got a responsibility of ensuring that they screen each and every patient who comes to the facility. For mental health illness.*” (DPM 1)

“*We’ve got the screening tool. We’ve got the national screening tool, the PC101, that the nurses at the clinic utilize for screening of all patients. So, it is a standardized tool that they use … The screening is done by the professional nurses at the facility level … every, eh, outpatient clinic should also conduct screening. Every professional there*.” (DPM 2)

Though DPMs indicated that screening should be available for all patients, some shared that they were still struggling to implement universal screening in all clinics.

“*I found that there are a few mobile clinics who are—still has a zero screening, and some of the other clinics have low screening, some have high screening, some have up to fifty percent, eighty percent that they are screening.*” (DPM 6)

#### Procedures for stepped-care referrals to mental healthcare practitioners and outside facilities

DPMs described various strategies for referral of patients after initial evaluation for mental illness by nurses. When a clinic has a full-time MHP located on-site, DPMs indicated that nurses should refer directly to these individuals. Following within-clinic referral, DPMs reported that an MHP is to conduct an assessment to diagnose the patient. Then, the MHP should either treat the patient or refer to another clinic for specialized care, depending on case severity.

“*If [the nurse] find [s] any “yes” answer on the screening tool, then [they] are supposed to refer this to a mental health practitioner. [The] mental health practitioner is usually the clinic sister who has training in psychiatric nursing. Then she can do a proper screening. There is a screening, assessment questionnaire that we have that she can use to do the assessment and come to a diagnosis, a nursing diagnosis. And then if she feels this person needs to be referred, sometimes she, she can either do some counselling with this person herself, if it’s manageable, or she can—If she thinks this person needs medication, she can refer this person to the nearest hospital where there is an outpatient clinic where psychiatric patients are seen.*” (DPM 6)

Even if a clinic did not have a MHP working full-time, DPMs explained that there were rotating MHPs available for onsite referral at some clinics.

“ *… most clinics do have visiting doctors, which are GPs that come and visit— [the nurse] book [s] the patient for the GP, to be seen. If it’s necessary for a psychologist or a social worker to see, depending on what the problems are, there are visiting psychologists also to the clinics. And [there are visiting] occupational therapists, as well.*” (DPM 6)

Following a diagnosis, next level referrals varied based on both clinic and patient factors. When patients suffered from more severe signs and symptoms—such as psychosis, violence/aggression, suicide intent/attempt—or the clinics did not have appropriate resources to manage patients onsite, DPMs indicated that these patients should be referred to a hospital. However, when social support and patient stability allowed, patients may be referred directly for outpatient care, ranging from clinic appointments to support group meetings.

*“ … depending on the condition of the patient, we also have a system where you phone the hospital to indicate that you’ve got this type of patient presenting with this. Because sometimes you’ll find someone, they have been on medication, but they’ve defaulted and then the hospital verifies, “Outpatient days for mental health patients are on this day,” especially if the patient has a support structure, like a caregiver or somebody with them, then you are able to book for that patient for that particular day.*” (DPM 1)

“*Support groups for the depressed and the anxious. Because we’ve got, uh, abused women who’ll be, eh, maybe depressed or be anxious, anxiety disorder, be—Uh, not at ease to be in the surrounding of other people because of the circumstances at home. So, they would refer to the women who experience the same who can share their experiences and maybe comfort and reassure that there is still life after what, whatever happened to you.*” (DPM 9)

While DPMs described hospital referral of patients already diagnosed with mental illness, this was only possible in clinics with a full-time or visiting MHP. In the clinics that did not have a MHP, most DPMs indicated that, after screening by a nurse, patients should be referred to specialized care in a different facility for diagnosis, often during an involuntary hold.

“*In fact, when it comes to mental health patients, all of them are referred to the next level of care for diagnosis unless we have, um, a psychologist or psychiatrist doing outreach services to that particular facility*. *Now, the most common process that is, is the seventy-two hour assessment procedure, whereby patients who are said to be mentally ill are then being admitted in the hospital forcefully or without a consent for assessment and care. Now, that is the process that we are using, in terms of diagnosing people with, eh, mental illness or who are assumed to have—to be suffering from mental illness.*” (DPM 2)

However, one DPM indicated that even in clinics where MHPs diagnose mental illness, confirmation of the diagnosis must first occur at the hospital level before proceeding with treatment.

“*…the standard operating procedures, as well as, uh, legislation, like the Mental Healthcare Act that, um, guides as well as to how we diagnose, um, mental healthcare patients and come up with a diagnosis for them. But you know that at primary healthcare level, it’s only a provisional diagnosis, and then the patient is normally being referred to the next level of care for the final diagnosis. And the patient is then down-referred back to primary healthcare for management.*” (DPM 1)

“Down-referral” back to the original clinic that referred the patient for specialized care was often discussed as a way to improve treatment continuity and outpatient management. In some cases, as described by DPM 1 above, down-referral occurs immediately after diagnosis. In other cases, DPMs described down-referral of patients that had first been treated at a secondary or tertiary care center.

*“ … if this person is better after treatment, [the hospital] discharges them, and either see them for a while at the outpatient department, and after they’ve stabilized well, they send them back to the clinics with the prescription sheet.”* (DPM 6)

To support care continuity, some DPMs indicated that down-referral should be accompanied by a treatment plan that clinic physicians and psychiatrists, if possible, would also review.

“*In other words, they down referred, maybe from the hospital to the clinic with the full package of treatment that they need to follow for this specified period of time. And then the medication is reviewed by a medical practitioner. Uh, preferably a psychiatrist.*” (DPM 1)

### Challenges to implementation of integrated mental health services in primary care

#### Absence of inter-program coordination at the district administrative level

Despite the proposed structure for screening, diagnosis, and patient management in primary care, DPMs identified several obstacles to the integration of mental healthcare into TB and MCH services. First, though there were great efforts to integrate primary and mental healthcare at the clinic level, most DPMs noted that programs were not being integrated on the administrative level within district management. DPMs expressed frustration that program administration remains siloed, with very little inter-program collaboration. They further explained that this fragmentation caused clinic staff to feel stressed by competing interests and directives from different programs.

“*Every program is focused on itself. There is very little talk between programs. So, from the top level, it’s already been separated, these programs, so it’s actually a pity that they have separated it so because it’s now difficult to come down and bring them all together because that’s what needed at the prim- at the gr- at the root level, grassroot level. You need to integrate the clinics. That’s what frustrates the clinic staff so much because you have so many programs, and um, they have to comply with every program and, for themselves, they don’t talk to each other. It’s like the programs—Each program is important.*” (DPM 2)

##### Lack of material and human resources

DPMs also reported a need for increased resources to support further integration in clinics. Most cited shortages in personnel and training opportunities, both of which often corresponded with a lack of funding. One DPM lamented the fact that resources have held her/his district back from creating a multidisciplinary mental health team to support implementation:

“*Psychiatrists, psychologists, occupational therapy, social worker, and a mental health nurse, and you know, if I had, we had the resources, we could already have appointed the whole team … … you know, to do training not only for nursing staff.*” (DPM 8)

Another summed up the other DPMs’ concerns concisely in explaining that the problem is human resources, material resources, and funding all combined:

“*And when I say strengthening mental health at sub-district and facility level, I mean both in terms of resources, human and material resources. And, um, that comes with a budget, of course.”* (DPM 2)

##### Low mental health literacy in both the district administration and general population

Additionally, most DPMs highlighted a need for increased mental health literacy and awareness among district-level administrators to better facilitate mental healthcare integration. A TB program manager stated that they had never been shown data on correlations between TB and mental health, which would promote collaborations between the TB and mental health programs and assist in prioritization of services. Some DPMs also explained that it would be helpful to track the screening and referral processes to provide feedback on how well primary care integration of mental health services was improving treatment for patients with mental illness.

“*We cannot assess the impact of this process- this screening … As an indicator we are recording, the number of patients screened. But we don’t know how many patients af- who have been screened, who have been found to have a problem and referred accordingly. So that is where the whole indicator is lacking*.” (DPM 2)

Along with improved mental health literacy at the district administration level, some DPMs emphasized a need to promote mental health awareness among the general population. They noted that introducing the notion that mental health is an integral part of overall health was critical to the success of integration efforts.

“*We need to, um, advocate more for it, you know. And start, you know, changing people’s mindset about where mental health needs to be, so you know, a lot of education, a lot of training*.” (DPM 8)

“*People never give me th- or rarely give me the healthy part of it … They give you the disease part of it … they neglect. The health part of it is not seen properly … I think maybe motiv- through the radio, through mass media, more awareness can be created, because everybody has got a mental health part of themselves.*” (DPM 6)

### Nurse mental health training, experience, and patient management

Quantitative questionnaires were administered to assess training on, experience with, and delivery of mental health services from the provider perspective. Forty-six (78.0%) nurses reported receiving some form of training on mental healthcare, though only 37 nurses (62.7%) reported receiving training on the PC101*.* Thirty-six (61.0%) nurses responded that the PC101 guidebook is “very easy” to access in the clinic. Over 75% (*n* = 45) of nurses reported seeing few or no patients with mental illness, though the majority (*n* = 35, 59.3%) responded they felt “very comfortable” or “comfortable” with the management of mental illness in patients (Table 3).

**Table 3:** Nurse mental health training and experience

Questionnaire response	N (%)
Any mental health training	
Yes	46 (78.0)
No	13 (22.0)
No response	0
PC101 training	
Yes	37 (62.7)
No	19 (32.2)
Don’t know	2 (3.4)
No response	1 (1.7)
PC101 accessibility	
Very easy	36 (61.0)
Easy	17 (28.8)
Difficult	1 (1.7)
Very difficult	1 (1.7)
Don’t know/No opinion	1 (1.7)
No response	3 (5.1)
Patients suspected of mental illness	
All	0
Many	0
Some	11 (18.6)
Few	30 (50.8)
None	15 (25.4)
Other	1 (1.7)
Don’t know	2 (3.4)
No response	0
Comfort level managing mental illness	
Very comfortable	5 (8.5)
Comfortable	30 (50.8)
Somewhat comfortable	13 (22.0)
Not at all comfortable	8 (13.6)
Don’t know	3 (5.1)
No response	0

Procedures used by nurses to screen and manage mental illness is reported in Table 4. All nurses (*n* = 59) reported having ever screened patients for mental illness. Most (*n* = 51; 86.4%) reported having screened for mental illness in the past three months, and 43 (72.9%) reported screening patients at every visit, though only 26 (44.1%) reported using a specific screening tool (e.g. the PC101). When a patient screened positive, most nurses (*n* = 34, 57.6%) reported they refer the patient to another practitioner within their clinic, of which 88.2% (*n* = 30) were MHPs.

**Table 4:** Screening and referral by nurses

Questionnaire response	N (%)
Screened a patient for a mental illness in the past 3 months?	
Yes	51 (86.4)
No	7 (11.9)
Don’t know	0
No response	1 (1.7)
Screen patients at every visit	
Yes	43 (72.9)
No	16 (27.2)
No response	0
Method of assessment for mental illness	
Structured/existing tool	26 (44.1)
Questions asked, no structured tool used	28 (47.5)
Clinical observation	4 (6.78)
Other	1 (1.7)
Don’t know	0
No response	0
Next steps after positive screen	
Managed/treated by nurse at clinic	2 (3.4)
Referred to someone else inside clinic	34 (57.6)
Referred to other healthcare facility	21 (35.6)
Don’t know	1 (1.7)
No response	1 (1.7)
Type of in-clinic practitioner patients referred to	
Mental health practitioner	30 (88.2)
Other	4 (11.8)

### Mental health practitioner management of referred cases

Fourteen (82.4%) MHPs reported that their responsibilities include assessing mental illness—13 (76.5%) indicated they screen for mental illness and seven (41.2%) indicated they diagnose mental illness. Five (35.7%) of the 14 MHPs who assess mental illness reported using a structured or semi-structured tool, two of whom reported the use of structured or semi-structured tools in combination with clinical observation. Of the 13 MHPs who screen patients, one (7.7%) reported treating and eight (57.1%) reported referring patients to another healthcare facility after a positive screen. Of the seven MHPs who diagnose patients, three (42.9%) reported treating and three (42.9%) reported referring patients they diagnose with mental illness. Regardless of whether they personally screen or diagnose patients, 14 (82.4%) of the 17 MHPs reported that they offered some form of treatment to patients with mental illness: seven (50.0%) reported prescribing psychiatric medications, four (28.6%) offered individual therapy, one (7.1%) offered group therapy, and two (14.3%) offered other treatment. Table 5 outlines MHP responses to questions on their role in the management of mental illness.

**Table 5:** Mental health practitioner management of mental illness

Questionnaire response	N (%)
Method of assessment for mental illness (*n* = 14)	
Structured/existing tool	5 (35.7)
Questions asked, no structured tool used	3 (21.4)
Clinical observation	5 (35.7)
Other	3 (21.4)
Don’t know	0
No response	1 (7.1)
Next steps after positive screen (*n* = 13)	
Treat	1 (7.7)
Refer	8 (61.5)
Other	1 (7.7)
No response	3 (23.1)
Next steps after positive diagnosis (*n* = 7)	
Treat	3 (42.9)
Refer	3 (42.9)
Other	1 (14.3)
No response	0
Type of treatment offered (*n* = 14)	
Psychiatric medications	7 (50.0)
Individual therapy	4 (28.6)
Group therapy	1 (7.1)
Other	2 (14.3)

## Discussion

Our mixed-methods findings indicate that while integration of mental healthcare into primary care clinics has been achieved to some degree, significant challenges remain in implementation of these efforts. DPMs outlined the responsibility for nurses to screen all patients in their care for mental illness. However, though all nurses reported some screening of patients, nurse and DPM responses indicated that screening was not universally implemented for all patients. Furthermore, nurses reported inadequate training on screening procedures. Additionally, though DPMs indicated a clear responsibility for MHPs in diagnosis, treatment, and referral of patients, not all MHPs reported engaging in these activities. In describing challenges related to integration efforts, DPMs highlighted insufficient funding and material resources, poor coordination at the district administrative level, and low mental health awareness of district administration and the general population.

Though DPMs indicated that nurses are responsible for screening all patients at the clinic, only 73% of nurses reported screening at every patient visit. Over 20% of nurses reported never receiving any training to provide mental health services, and only 44% reported using a specific screening tool. Moreover, 32% of nurses reported never being trained to administer the PC101, the diagnostic screening tool intended for nationwide use in integrated mental health and primary care settings. Similar to our findings, previous research in other regions of South Africa has highlighted lack of training as a significant shortcoming in current integration efforts [40–43]. As nurses serve as the gateway to mental healthcare in primary care settings, future success of integration efforts depends on improving nurse training and the identification of implementation strategies [44] to facilitate consistent screening of patients by nurses.

After screening by nurses, DPMs described a variety of approaches to providing stepped care for patients depending on patient and clinic factors. Most DPMs indicated that in clinics with full- or part-time MHPs, nurses screen patients for mental illness and refer patients that screen positive to in-clinic MHPs for diagnosis. MHPs diagnose and then either treat the patient or refer for more specialized care. In clinics lacking MHPs, nurses screen patients for mental illness and refer patients that screen positive to another facility for diagnosis and treatment. While the different referral procedures reported present flexible alternatives for managing patients depending on clinic resources and case severity, past research has indicated challenges associated with these practices in South Africa. Specifically, South African hospitals to which patients are referred for 72-h observation are reported to have inadequate infrastructure, trained specialist personnel, and appropriate space for caretaking of patients with mental illness [18, 45–47]. Additionally, in a study conducted in a different district of South Africa, the use of down referral of patients to primary health clinics after diagnosis and/or treatment in specialized care was found to be associated with challenges in medication management and continuity of care [40]. However, this study also found that primary care practitioners felt comfortable with stable patients who were down-referred. To improve this pathway in stepped care, a clear protocol for down-referrals that promotes the transfer of stable patients with proper documentation of prescribed medications and follow-up care must be developed.

District managers expressed clear roles for MHPs in the diagnosis, treatment, and referral of mental health patients. Despite this articulation, not all MHPs reported engaging in these activities. For example, 12% of MHPs indicated they did not diagnose, treat, or refer patients with mental illness. The difference in MHPs’ role in patient management is likely owing to the non-uniform implementation of screening, treatment, and referral processes. A qualitative study conducted with health administrators in a different district of South Africa identified similar heterogeneity in procedures and roles across clinics. These inconsistences in implementation were attributed to insufficient training and resources, poor communication about the policy framework, and poor coordination of planning between national, provincial, and district levels [41].

In the present study, DPMs reported similar challenges to implementing integrated mental healthcare services with other program areas. Many DPMs mentioned inadequate funding for mental health, and the subsequent lack of trained personnel and clinic infrastructure. Currently, South Africa spends 5% of the health budget on mental health [48], but evaluations of economic burden of mental illness and cost effectiveness of service scale-up indicate that the total costs of untreated mental illness far outweigh the proposed treatment costs. A recent study estimated the total lost earnings associated with depression and anxiety in South Africa amount to $3.6 billion annually, whereas government spending at the time was estimated to be just $59 million [49]. Moreover, the total cost of delivering South Africa’s proposed intervention package at target coverage levels was estimated to be only $1.86 per capita, and only $0.16 per capita would need to be invested each year to achieve target coverage levels in 5 years [50]. A recent review indicated mental health integration into primary care and community services is the most cost-effective method to increase access to mental health services in South Africa [51]. Consequently, it is fundamental that the funding gap in current integration efforts be addressed.

DPMs in our study also cited poor communication and coordination at the administrative level as barriers to integration and implementation. Previous studies from South Africa have described the impact of poor inter-sectoral planning at the national level filtering down to provincial and district levels and creating confusion around mandates and roles [41, 43]. While this remains a challenge across LMICs [52], strategies have been identified at national policy levels to promote district administrative collaboration and facilitate engagement at the clinic level. Such strategies include: outlining a shared vision across programs, clarifying roles and responsibilities of each program, establishing formal channels to facilitate program interaction at the national through district level, and facilitating mental health awareness efforts for administrators of other programs [43, 53, 54].

District managers also noted a need to improve monitoring and evaluation of integration progress to promote mental health awareness. South Africa has strong policy and infrastructure on district health information management systems relative to other LMICs. However, the current district health information system collects data on a very limited range of mental health program indicators, none of which are sufficient to determine if diagnosis and management of mental illnesses are being achieved [12, 55]. To ensure that increased efforts to integrate mental healthcare are successful, a broader range of indicators—such as specific diagnoses and severity, rates of engagement in care, retention in treatment, and psychiatric inpatient bed occupancy—must be adopted and implemented [56].

In the midst of our data collection, a very high profile event involving the death of at least 94 mental health patients occurred in Gauteng Province [57], where one of our study districts was located. This event, referred to as the “Life Esidimeni Tragedy,” occurred after the Gauteng Department of Health canceled a contract with a mental healthcare provider, Life Esidimeni, and transferred 1371 mental health patients to multiple NGOs across the province. The official government report determined that many of the NGOs “were poorly resourced, had neither the basic competence or experience, nor the leadership/managerial capacity or fitness for purpose” to take on these patients. This ill-prepared shift in mental healthcare provision, and consequential loss of life, has shone a spotlight on public sector mental health services and needs in South Africa. Therefore, this moment provides a critical opportunity to highlight the progress and barriers toward integrating mental health services into primary health services as a means to effectively deinstitutionalize and decentralize mental healthcare.

### Limitations

While this study provides important insights on mental healthcare practices and management within primary care in South Africa, there are several limitations. The high profile “Life Esidimeni Tragedy” could have caused an abnormal increase in mental health awareness, and participant responses may not be representative of past procedures in patient management. Additionally, purposive selection of clinics may have introduced bias, as the study team and participating districts identified rural and urban clinics subjectively rather than using a specific metric to define these regions. Moreover, MHPs come from a variety of professional backgrounds (e.g. psychiatrist, social worker, nurse, etc.) which may influence their comfort managing mental illness and, in turn, the activities they perform in the management of mental illness. However, we did not collect information on professional background from study participants, so we were unable to explore this potential association. Finally, future research should include more extensive, qualitative interviews with both nurses and MHPs to further triangulate and compare in-depth findings from DPM interviews.

## Conclusions

Across four districts in South Africa, we found that integration of mental health services has been implemented to some degree in TB and MCH services of primary care clinics. However, there is a considerable lack of training and consistency in the uptake of roles and responsibilities by nurses and mental health practitioners. Improved district-level administrative coordination, mental health awareness, and financial resources are critical to the success of current integration efforts. Given the Life Esidimeni Tragedy, there is currently a national dialogue regarding the care and management of mental illness in South Africa. As such, the time is ripe to advocate for strengthened efforts toward the integration of mental healthcare services into primary care to achieve The South African National Mental Health Policy Framework and Strategic Plan goals by 2020.

## Abbreviations

DPM: District program manager
LMICs: Low- and middle-income countries
MCH: Maternal child health
MHP: Mental health practitioner
PC101: Primary Care 101 Guidelines
TB: Tuberculosis
WHO: World Health Organization
